# Systemic Lupus Erythematosus Exacerbates Hip Arthritis by Promoting Chondrocyte Pyroptosis in the Femoral Head via Activating the NF‐κB Pathway

**DOI:** 10.1111/jcmm.70531

**Published:** 2025-04-03

**Authors:** Xuliang Fang, Helou Zhang, Huiqing Zhou, Shuchao Shen, Zhaobai Lao, Zhiguo Zhang, Yishan Bian, Chengcong Zhou, Hongting Jin, Peijian Tong, Yanqun Huang, Hong Zhou, Hanbing Zeng, Fangda Fu, Chengliang Wu, Wenbiao Zheng, Hongfeng Ruan

**Affiliations:** ^1^ Institute of Orthopaedics and Traumatology The First Affiliated Hospital of Zhejiang Chinese Medical University (Zhejiang Provincial Hospital of Traditional Chinese Medicine) Hangzhou China; ^2^ Department of Orthopaedics The First Affiliated Hospital of Zhejiang Chinese Medical University (Zhejiang Provincial Hospital of Traditional Chinese Medicine) Hangzhou China; ^3^ Hangzhou Fuyang Hospital of TCM Orthopedics and Traumatology Hangzhou China; ^4^ The Second Clinical Medical College, Zhejiang Chinese Medical University Hangzhou China; ^5^ Department of Orthopedics Taizhou Municipal Hospital Taizhou China

**Keywords:** articular cartilage degeneration, chondrocyte pyroptosis, hip arthritis, NF‐κB pathway, systemic lupus erythematosus

## Abstract

Systemic lupus erythematosus (SLE) is an autoimmune disease characterised by chronic inflammation and immune dysregulation, significantly impacting multiple organ systems, including the joints. While SLE is known to contribute to musculoskeletal complications, its role in hip arthritis development and the underlying mechanisms remain poorly understood. This study aims to investigate the relationship between SLE and hip arthritis progression using MRL/*lpr* mice, which exhibit early‐onset SLE, compared with MRL/*MpJ* control mice at 14 weeks of age. Through comprehensive histological, immunohistochemical and molecular analyses, we evaluated articular cartilage (AC) degeneration, extracellular matrix (ECM) metabolism, inflammatory responses, and chondrocyte pyroptosis. Our results demonstrated that MRL/*lpr* mice developed an accelerated hip arthritis‐like phenotype, manifesting as enhanced AC degeneration, impaired chondrocyte proliferation, heightened apoptosis and promoted inflammatory cytokine production. Notably, SLE markedly stimulated chondrocyte pyroptosis by increasing pyroptosis‐related proteins, including NLRP3, ASC, CASPASE‐1 and GSDMD, via activating the NF‐κB pathway. These findings establish a novel mechanistic link between SLE and hip arthritis progression, demonstrating that SLE promotes chondrocyte pyroptosis to exacerbate AC degeneration via NF‐κB activation, highlighting chondrocyte pyroptosis as a key driver of SLE‐associated hip arthritis and a potential therapeutic target for mitigating SLE‐induced joint manifestations.

## 1 Introduction

Systemic lupus erythematosus (SLE) is a complex autoimmune disease characterised by immune dysregulation, chronic inflammation and the production of autoantibodies, significantly affecting multiple organ systems, particularly in women of reproductive age [1, 2]. The formation of immune complexes and resulting inflammatory responses in SLE drive widespread organ damage, including the kidneys, skin, central nervous system and skeletal system [3, 4]. Among these manifestations, musculoskeletal involvement, particularly arthralgia, affects over 80% of SLE patients and is often one of the earliest symptoms [5, 6, 7]. Despite the well‐documented effects of SLE on joints, the specific mechanisms by which SLE exacerbates arthritis progression, particularly in the hip joint, remain poorly understood.

Arthritis is a major cause of joint pain and disability, with the progressive degeneration of articular cartilage (AC) as its hallmark feature. The degeneration is primarily driven by disruptions in extracellular matrix (ECM) metabolism, including the degradation of key structural components like collagen type II (COL2) and AGGRECAN [8, 9] and the upregulation of ECM‐degrading enzymes, such as metalloproteinase (MMP) 3 and MMP13, as well as AGGRECANase, ADAMTS‐5. Additionally, impaired proliferative capacity and increased apoptosis of chondrocytes contribute to the reduced number of functional chondrocytes, accelerating cartilage damage [10]. Inflammation further exacerbates this degeneration, as inflammatory cytokines, such as interleukin‐1 beta (IL‐1β), IL‐6, IL‐18 and tumour necrosis factor‐alpha (TNF‐α), promote ECM degradation, highlighting inflammation as a critical driver of arthritis pathogenesis [11, 12, 13]. Interestingly, emerging evidence from SLE patients has identified elevated levels of pro‐inflammatory cytokines, including IL‐1β, IL‐18 and TNF‐α in their serum [14, 15, 16], while our latest findings from SLE model mice have found significant elevation of these inflammatory cytokines within intervertebral disc, which are implicated in organ damage and the development of inflammatory arthritis in these tissues [17]. This raises the question of whether SLE‐induced inflammatory stimuli disrupt chondrocyte activity, leading to AC degeneration in the hip joint.

Pyroptosis is a form of programmed cell death distinguished by its inflammatory nature, involving nod‐like receptor protein‐3 (NLRP3) activation, CASPASE‐1‐mediated cleavage of gasdermin D (GSDMD) and release of IL‐1β and IL‐18 [18, 19]. The NLRP3 inflammasome plays crucial roles in both the innate and adaptive immune systems, contributing to various autoimmune diseases, such as SLE and its induced joint pathologies [20]. Previous studies have demonstrated that NLRP3 activation exacerbates inflammation and bone erosion in SLE‐associated rheumatoid arthritis [21], while pyroptosis‐associated factors, such as GSDMD, are highly expressed in renal tubules and peripheral blood mononuclear cells (PBMCs) of SLE patients and lung tissues of SLE‐PAH mice [22, 23, 24]. Given the clear role of NLRP3‐mediated pyroptosis in the SLE pathophysiology and its associated complications and the high expression of pyroptosis inflammatory effectors IL‐1β and IL‐18 in SLE patients, it is reasonable to conclude that chondrocyte pyroptosis may be involved in the pathological process of SLE‐induced hip AC degeneration and arthritis progression.

The nuclear factor kappa‐Β (NF‐κB) pathway is a key regulator of inflammation and significantly influences the development of autoimmune diseases [25] and arthritis [26]. It consists of five members: NF‐κB1 (P50), NF‐κB2 (P52), RelA (P65), RelB and c‐Rel. Under normal conditions, inactive P65 dimers are sequestered in the cytoplasm by an inhibitor of κB (I‐κBα) [27]. Upon stimulation by inflammatory mediators, I‐κBα is phosphorylated, facilitating the release and nuclear translocation of P65, which leads to the upregulation of genes involved in inflammation, such as inducible nitric oxide synthase (iNOS) and cyclooxygenase‐2 (COX‐2), along with MMPs that are contributing to ECM degradation, chondrocyte apoptosis and cartilage inflammation [28, 29]. Extensive research has confirmed the association of the NF‐κB pathway with SLE pathogenesis, especially in SLE‐associated nephritis and neuropsychiatric lupus, where therapeutic targeting of NF‐κB has shown efficacy [30, 31, 32]. However, it remains to be determined whether SLE deterioration aggravates chondrocyte pyroptosis by activating the NF‐κB pathway, thereby promoting AC degeneration and hip arthritis progression.

Given the prevalence of SLE‐associated arthritis, the lack of effective treatment options and the importance of early diagnosis and treatment concepts, we aimed to investigate the potential pathogenesis by which SLE exacerbates AC degeneration and hip arthritis progression. Using MRL/*lpr* lupus‐prone mice and their control counterparts, MRL/*MpJ* mice, we employed morphological staining, immunohistochemical analysis and TUNEL assays to evaluate the complex interplay between SLE and AC degeneration. Our findings contribute to a novel understanding of SLE in vivo as a core driver of AC degeneration and hip arthritis, offering insights into potential therapeutic strategies for SLE‐associated arthritis.

## 2 Materials and Methods

### 2.1 Chemicals and Reagents

Primary antibodies against COL2, MMP3, MMP13, PCNA, KI67, BCL‐2, BAX, TNF‐α, P65 and phospho‐I‐κBα (p‐I‐κBα) were obtained from Ruiying Biological Co. (Jiangsu, China). Antibodies for AGGRECAN, ADAMTS‐5 and GSDMD were acquired from Abcam Company Ltd (Cambridge, MA, USA). IL‐1β, IL‐18 and ASC antibodies were supplied by Bioss (Beijing, China), while antibodies against CASPASE3, IL‐6, iNOS, COX‐2, NLRP3 and CASPASE‐1 were from Proteintech (Wuhan, China). Unless otherwise specified, all other reagents were obtained from Sigma‐Aldrich (St. Louis, MO, USA).

### 2.2 Animals and Experimental Design

MRL/*lpr* mice are invaluable models for investigating both SLE and its associated manifestations, characterised by high circulating autoantibody concentrations and immune complex deposition, mimicking human SLE due to defective Fas signalling [33]. MRL/*MpJ* mice, carrying the functional Fas signalling, serve as controls with delayed and milder onset of SLE symptoms [34]. Specifically, MRL/*MpJ* mice typically exhibit SLE symptoms around 24 weeks of age, while MRL/*lpr* mice develop SLE earlier at approximately 12 weeks [35].

Consequently, 6‐week‐old female MRL/*MpJ* and MRL/*lpr* mice were obtained from the Center Animal House of Zhejiang Chinese Medical University and housed under specific pathogen‐free conditions with controlled conditions (23°C ± 2°C), a 12‐h light/dark cycle and free access to water and standard lab chow. All protocols for mouse procedures were approved by the Committee on the Ethics of Animal Experiments of Zhejiang Chinese Medical University (No. IACUC‐20211101‐04).

At 14 weeks of age, all mice were euthanised and femoral heads were harvested for subsequent histopathological analysis. All animal experiments were obedient to the ARRIVE guidelines and conducted in accordance with the UK Animals (Scientific Procedures) Act, 1986 and related guidelines, EU Directive 2010/63/EU for animal experiments [36].

### 2.3 Histological Staining, Immunohistochemistry and Immunofluorescent

Femoral head samples were fixed in 4% paraformaldehyde for 48 h, decalcified with 14% EDTA solution for 21 days, and embedded in paraffin for sectioning at 5 μm. Sections underwent deparaffinisation and graded alcohol washes, followed by haematoxylin–eosin (H&E) staining and Safranin O/Fast green (SO/FG) staining. Structural cartilage changes were assessed using a modified OA Research Society International (OARSI) scoring system by two blinded observers, as previously described [26].

For immunohistochemistry (IHC), sections were rehydrated, subjected to antigen retrieval in 0.01 mol/L citrate buffer, and treated with 0.3% hydrogen peroxide to reduce endogenous peroxidase activity. Non‐specific staining was blocked with normal goat serum. Subsequently, the sections were incubated with primary antibodies, including COL2 (1:200), MMP3 (1:200), MMP13 (1:200), AGGRECAN (1:200), ADAMTS‐5 (1:200), IL‐1β (1:900) and IL‐18 (1:900), overnight at 4°C, followed by secondary antibody incubation for 30 min. Visualisation was achieved using diaminobenzidine solution (ZSGB‐BIO, Beijing, China), and sections were counterstained with haematoxylin. Negative controls were prepared by omitting primary antibodies. Integrated optical density (IOD) of antigen expression was quantified using Image‐Pro Plus 6.0 (Media Cybernetics, Silver Spring, MD, USA) in a blinded manner.

For immunofluorescent (IF) analysis, sections were incubated with primary antibody (1:500 dilution) against PCNA, KI67, CASPASE3, BCL‐2, BAX, IL‐6, TNF‐ɑ, iNOS, COX‐2, NLRP3, ASC, CASPASE‐1, GSDMD, P65 and p‐I‐κBα at 4°C overnight. Fluorescent secondary antibodies (Sungene Biotech, Tianjin, China) were applied for 30 min in the dark, followed by counterstaining with DAPI. Sections were imaged using a Carl Zeiss fluorescence microscope (Gottingen, Germany). Quantitative histomorphometric analysis of IOD was performed in a blind manner using the Image‐Pro Plus Software version 6.0.

### 2.4 TUNEL Staining

Chondrocyte apoptosis was examined using the TUNEL BrightGreen Apoptosis Detection Kit (Vazyme Biotech, Nanjing, China) according to the manufacturer's instructions. DAPI staining was used to estimate the total cell count, and sections were analysed using a fluorescence microscope (Carl Zeiss). The number of positive cells was quantified in six sections of each group.

### 2.5 Statistical Analysis

Data are presented as means ± SEM. Statistical analyses were performed using GraphPad Prism software 8.0 (San Diego, CA, United States). An independent‐sample *t*‐test was used for group comparisons, and differences were considered statistically significant at *p* < 0.05.

## 3 Results

### 3.1 SLE Deteriorates Hip Arthritis Progression in MRL/*lpr* Mice

**FIGURE 1:**
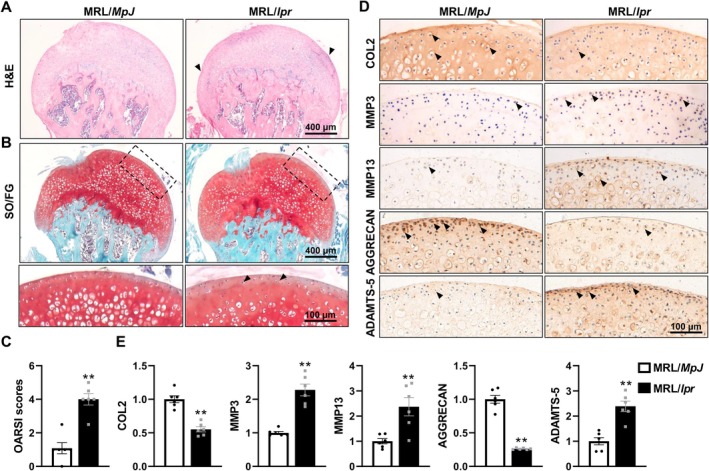
SLE promotes AC degeneration and ECM degradation in the femoral heads of MRL/*lpr* mice. (A, B) Representative images of H&E staining (A) and SO/FG staining (B) of femoral heads from MRL/*MpJ* and MRL/*lpr* mice. (C) Quantification of the severity of AC degeneration in the femoral heads is shown in A by the OARSI scoring system. (D, E) Representative images (D) and corresponding quantification (E) of ECM components (COL2, AGGRECAN) and ECM‐degrading enzymes (MMP3, MMP13 and ADAMTS‐5) (IHC staining) in the femoral heads. Triangles indicate the positive‐staining area. Each experiment was performed in triplicate. Data are expressed as the mean ± SEM. **p < 0.01 (vs. MRL/*MpJ* mice), *n* = 6 per group.

To gain a comprehensive understanding of the intricate relationship between SLE and hip arthritis, MRL/*MpJ* and MRL/*lpr* mice were bred, and the SLE phenotype in MRL/*lpr* mice was verified according to previously established biomarkers and histopathological criteria, as detailed in our earlier publication [17]. To investigate the impact of SLE on hip arthritis progression, the morphological changes of femoral heads were assessed using H&E staining and Safranin SO/FG staining. The results showed that MRL/*MpJ* mice maintained a smooth and continuous cartilage surface with uniform chondrocyte distribution and strong Safranin O staining, indicating robust proteoglycan content. In contrast, MRL/*lpr* mice displayed pronounced degenerative changes. H&E staining highlighted surface depressions in the femoral head cartilage, disrupting its structural integrity. SO/FG staining showed a marked reduction in cartilage matrix content, as evidenced by a significant loss of Safranin O staining intensity. These findings suggest that SLE accelerates structural deterioration and matrix depletion in the femoral head cartilage, contributing to early joint AC degeneration (Figure 1A,B). The quantitative arthritis scores further substantiated these observations, showing markedly higher OARSI scores in MRL/*lpr* mice compared to MRL/*MpJ* controls (Figure 1C), indicating more severe joint degeneration in SLE mice. Collectively, these results underscore that SLE accelerates hip joint degeneration by promoting cartilage structural damage and disrupting ECM content.

### 3.2 SLE Disrupts ECM Metabolism in the Femoral Heads of MRL/*lpr* Mice

Maintaining the homeostasis of ECM metabolism is critical for preserving chondrocyte function and adapting to external stress [8, 37]. To explore the effects of SLE on ECM metabolism in the femoral head, we determined the expression of key cartilage matrix components, COL2 and AGGRECAN, and their degrading enzymes (MMP3, MMP13 and ADAMTS‐5) using IHC analysis. The results indicated significant alterations in ECM metabolism in MRL/*lpr* mice (Figure 1D,E). Specifically, there was an approximately 50% reduction in COL2 expression and a 75% decrease in AGGRECAN. Conversely, the expression levels of MMP3, MMP13 and ADAMTS‐5 more than doubled in comparison to the MRL/*MpJ* controls. These findings further substantiate the deleterious effects of SLE on ECM metabolism in the affected joints.

### 3.3 SLE Impairs Proliferation and Enhances Apoptosis of Chondrocytes in the Femoral Heads of MRL/*lpr* Mice

**FIGURE 2:**
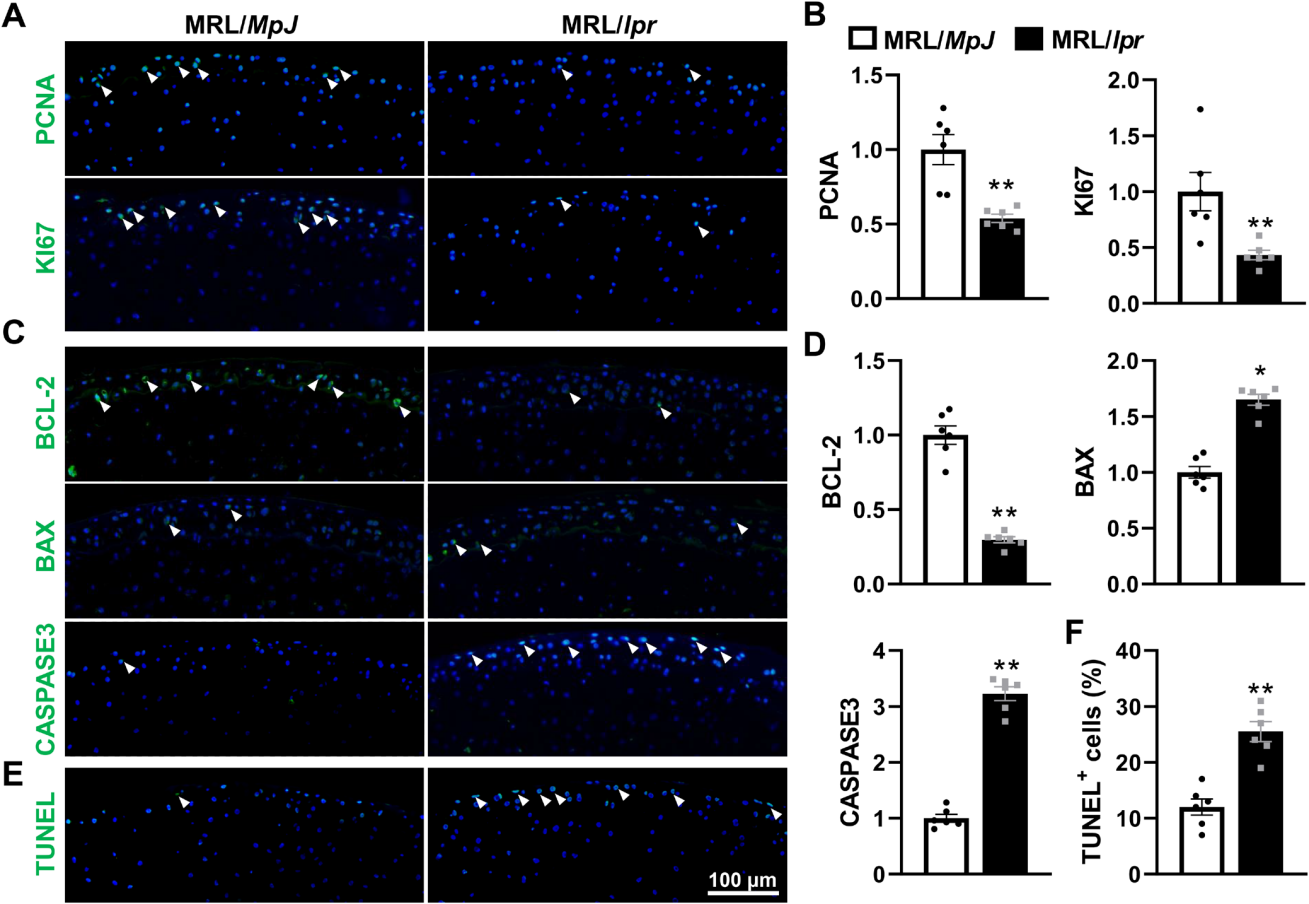
SLE suppresses chondrocyte proliferation while increasing apoptosis in femoral head AC of MRL/*lpr* mice. (A, B) Representative images (A) and corresponding quantification (B) of proliferation markers PCNA and KI67 (IF staining) in the femoral heads of MRL/*MpJ* and MRL/*lpr* mice. (C, D) Representative images (C) and corresponding quantification (D) of apoptosis‐related proteins (BCL‐2, BAX and CASPASE3) (IF staining) in the femoral heads. (E, F) TUNEL staining (E) and corresponding quantification (F) of TUNEL‐positive cells. Triangles indicate positive staining and DAPI stains nuclei (blue). Each experiment was performed in triplicate. Data are expressed as the mean ± SEM. *p < 0.01, **p < 0.01 (vs. MRL/*MpJ* mice), *n* = 6 per group.

To explore the impact of SLE on cellular dynamics, focusing on proliferation and apoptosis within chondrocytes. Our findings indicated significant suppression in cell proliferation of chondrocytes in the femoral heads of MRL/*lpr* mice, as evidenced by reduced expression of specific proliferation markers PCNA and Ki67, which decreased to 53% and 43% of the levels observed in MRL/*MpJ* controls, respectively, suggesting that SLE impairs the regenerative capabilities of chondrocytes in the femoral heads (Figure 2A,B). In addition, a significant disruption in the balance of apoptosis regulators was observed, with the anti‐apoptotic protein BCL‐2 reduced by 70% and the pro‐apoptotic protein Bax increased by 1.65‐fold, indicating a shift towards apoptotic processes in these mice (Figure 2C,D). This shift was further corroborated by a substantial elevation in CASPASE‐3 expression, a key executor of apoptosis, which aligns with the findings from the TUNEL assay showing increased DNA fragmentation in the MRL/*lpr* mice (Figure 2C–E). These results collectively demonstrate a compromised cellular environment in the femoral heads of MRL/*lpr* mice, characterised by reduced proliferation and enhanced apoptosis, contributing to the degenerative changes observed in the context of SLE.

### 3.4 SLE Promotes Inflammatory Cytokine and Enzyme Overexpression in Femoral Heads of MRL/*lpr* Mice

**FIGURE 3:**
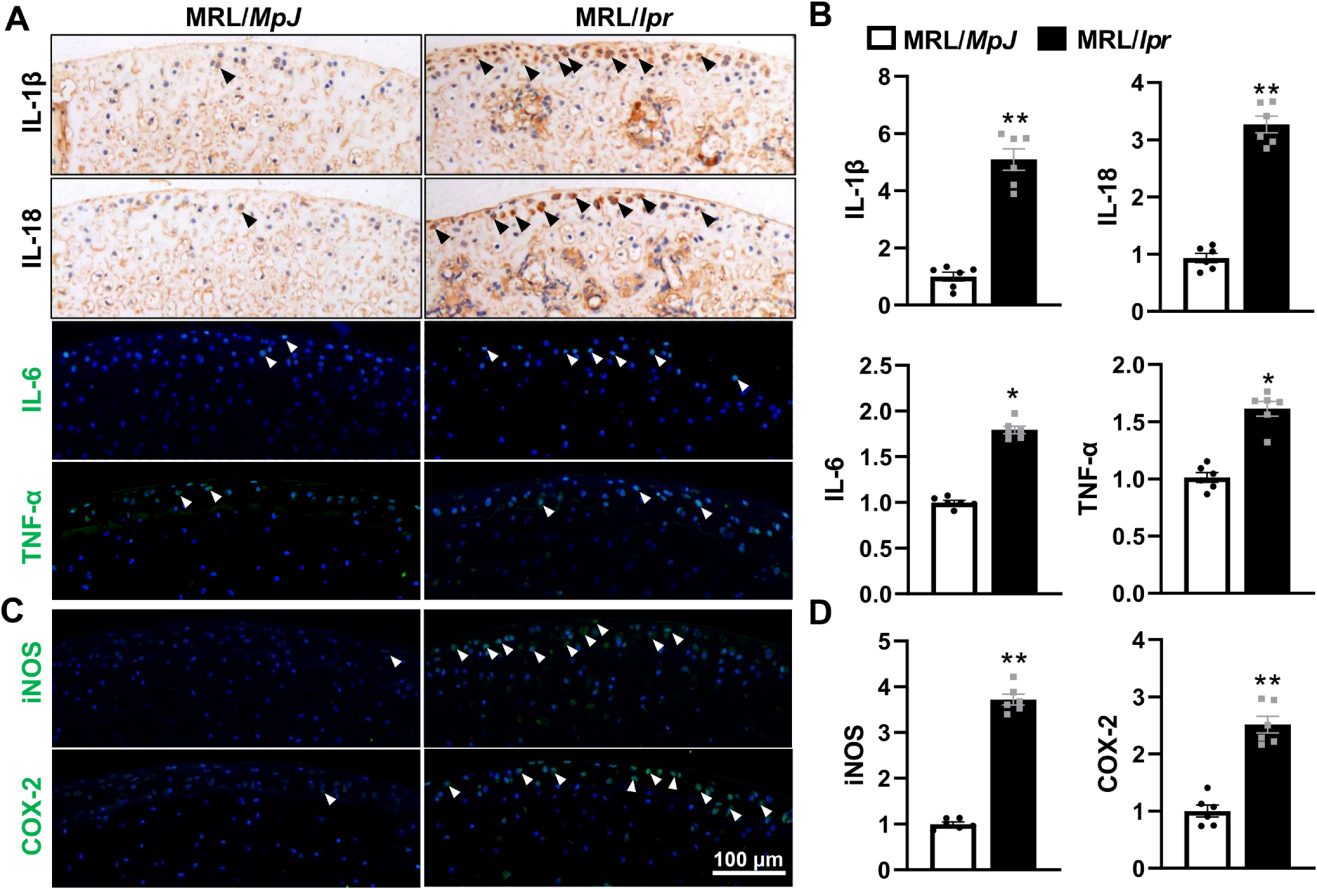
SLE enhances inflammation responses in the femoral heads of MRL/*lpr* mice. (A, B) Representative images (A) and corresponding quantification (B) of IL‐1β and IL‐18 (IHC staining), IL‐6 and TNF‐α (IF staining) expression in the femoral heads of MRL/*MpJ* and MRL/*lpr* mice. (C, D) Representative images (C) and corresponding quantification (D) of iNOS and COX‐2 (IF staining) in the femoral heads. Triangles indicated positive staining and DAPI stains nuclei (blue). Each experiment was performed in triplicate. Data are expressed as the mean ± SEM. *p < 0.05, **p < 0.01 (vs. MRL/*MpJ* mice), *n* = 6 per group.

Considering the established role of inflammatory cytokines in arthritis pathology [38], to understand the impact of SLE on inflammatory responses in the femoral heads of MRL/*lpr* and MRL/*MpJ* mice, we quantified the expression levels of inflammatory cytokines and enzymes, including IL‐1β, IL‐18, IL‐6, TNF‐α, iNOS and COX‐2 and found significantly elevated levels of IL‐1β and IL‐18 in MRL/*lpr* mice, showing approximately 5.1‐fold and 3.3‐fold increases, respectively, compared to MRL/*MpJ* controls (Figure 3A,B), indicating heightened inflammatory activity. Similarly, the expression of IL‐6 and TNF‐α, both potent mediators of inflammation, was also markedly increased by approximately 1.8‐fold and 1.6‐fold, respectively, in the SLE‐affected mice (Figure 3A,B). This pro‐inflammatory milieu was further supported by the upregulation of iNOS and COX‐2, enzymes that are crucial in the pathogenesis of inflammatory responses, with iNOS increasing by approximately 3.7‐fold and COX‐2 by 2.5‐fold in MRL/*lpr* mice compared to controls (Figure 3C,D). These results highlight a significant activation of inflammatory pathways in the femoral heads of MRL/*lpr* mice, demonstrating the profound impact of SLE on promoting local inflammation and potentially contributing to tissue degeneration.

### 3.5 SLE Promotes Chondrocytes Pyroptosis in Femoral Heads of MRL/*lpr* Mice

**FIGURE 4:**
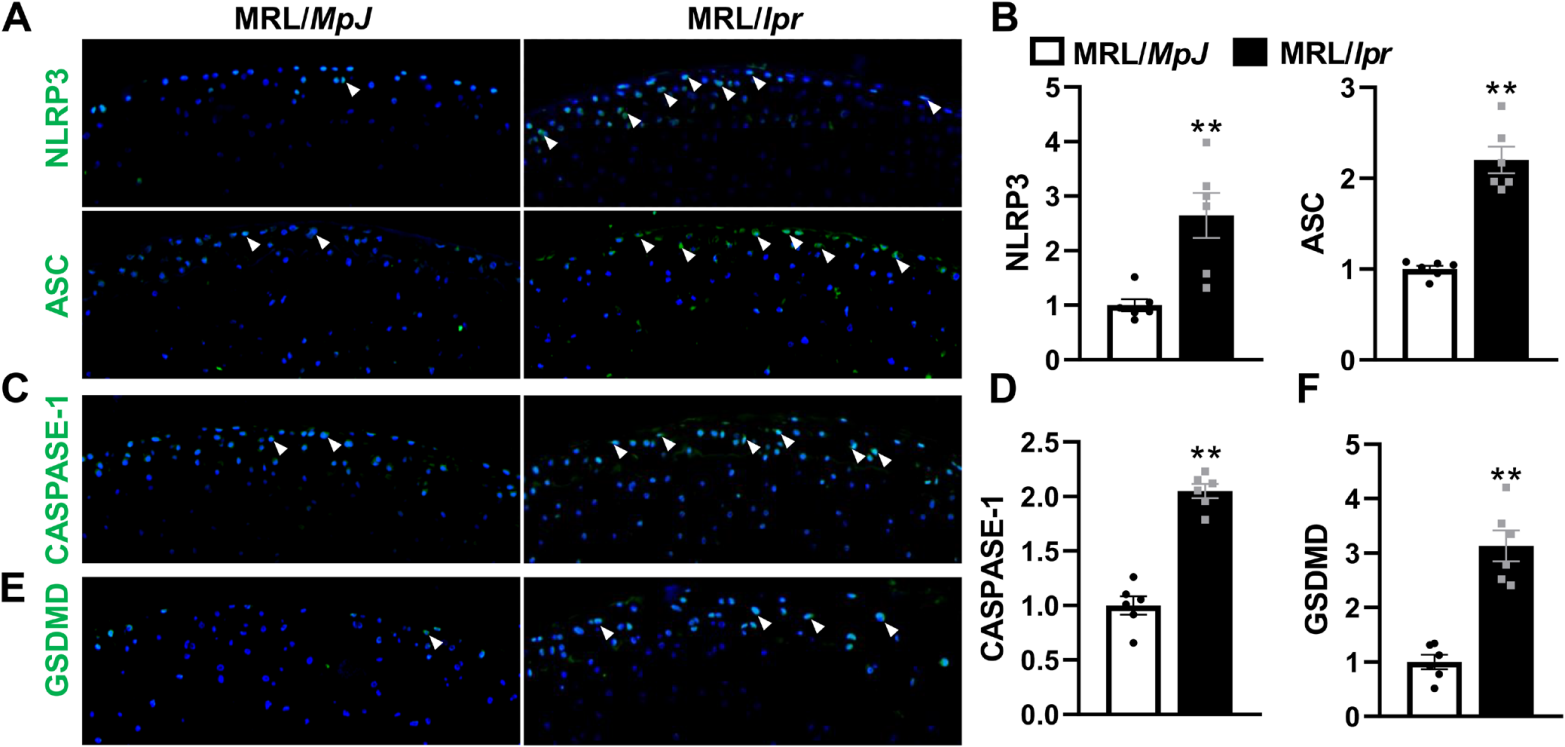
SLE promotes chondrocyte pyroptosis in chondrocytes of femoral heads of MRL/*lpr* mice. (A–F) Representative images (A, C, E) and corresponding quantification (B, D, F) of key pyroptotic factors including NLRP3, ASC (A, B), CASPASE‐1 (C, D) and GSDMD (E, F) (IF staining) in the femoral heads of MRL/*MpJ* and MRL/*lpr* mice. Triangles indicated positive staining and DAPI stains nuclei (blue). Each experiment was performed in triplicate. Data are expressed as the mean ± SEM. **p < 0.01 (vs. MRL/*MpJ* mice), *n* = 6 per group.

In view of the heightened expression of inflammatory factors, especially IL‐1β and IL‐18, which are key downstream effectors of pyroptosis, observed in the cartilage of SLE‐affected mice [18, 19], as well as in the progression of arthritis [39], we further investigated the activation of pyroptotic pathways in the cartilage of SLE‐affected mice by examining the expression of key pyroptosis‐related proteins, including NLRP3, ASC, CASPASE‐1 and GSDMD. IF staining results revealed a significant upregulation of NLRP3 and ASC in the AC of MRL/*lpr* mice, with NLRP3 expression increasing by approximately 2.6‐fold and ASC by 2.2‐fold compared to MRL/*MpJ* controls (Figure 4A,B). CASPASE‐1, a critical component of the inflammasome pathway, was also significantly elevated by 2‐fold, indicating enhanced inflammasome activation in SLE‐affected tissues (Figure 4C,D). Additionally, GSDMD, the executor of pyroptosis, was markedly upregulated, with a 3.1‐fold increase in MRL/*lpr* mice, further confirming the activation of pyroptotic cell death in these tissues (Figure 4E,F).

### 3.6 SLE Further Activates NF‐κB Signalling in Superficial Chondrocytes of MRL/*lpr* Mice

**FIGURE 5:**
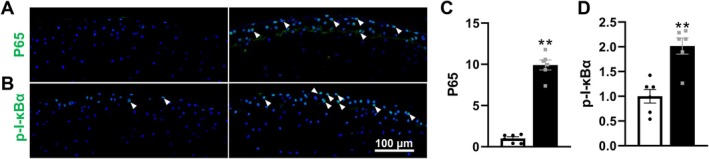
SLE activates the NF‐κB pathway in chondrocytes of the femoral head of MRL/*lpr* mice. (A–D) Representative images (A, B) and corresponding quantification (C, D) of P65 and p‐I‐κBα (IF staining) in the femoral heads. Triangles indicated positive staining and DAPI stains nuclei (blue). Each experiment was performed in triplicate. Data are expressed as the mean ± SEM. **p < 0.01 (vs. MRL/*MpJ* mice), *n* = 6 per group.

Given that the NF‐κB pathway serves as an upstream regulator of chondrocyte pyroptosis, we assessed its activation in the cartilage of SLE‐affected mice. IF revealed a striking 10‐fold increase in p65 expression in MRL/*lpr* mice (Figure 5A,B), accompanied by a 2‐fold increase in p‐IκBα, indicating robust activation of the NF‐κB pathway (Figure 5C,D). These findings demonstrate that the heightened NF‐κB activity in SLE‐affected cartilage may contribute to chondrocyte pyroptosis in the femoral heads of MRL/*lpr* mice, thereby exacerbating inflammation and AC degeneration.

## 4 Discussion

**FIGURE 6:**
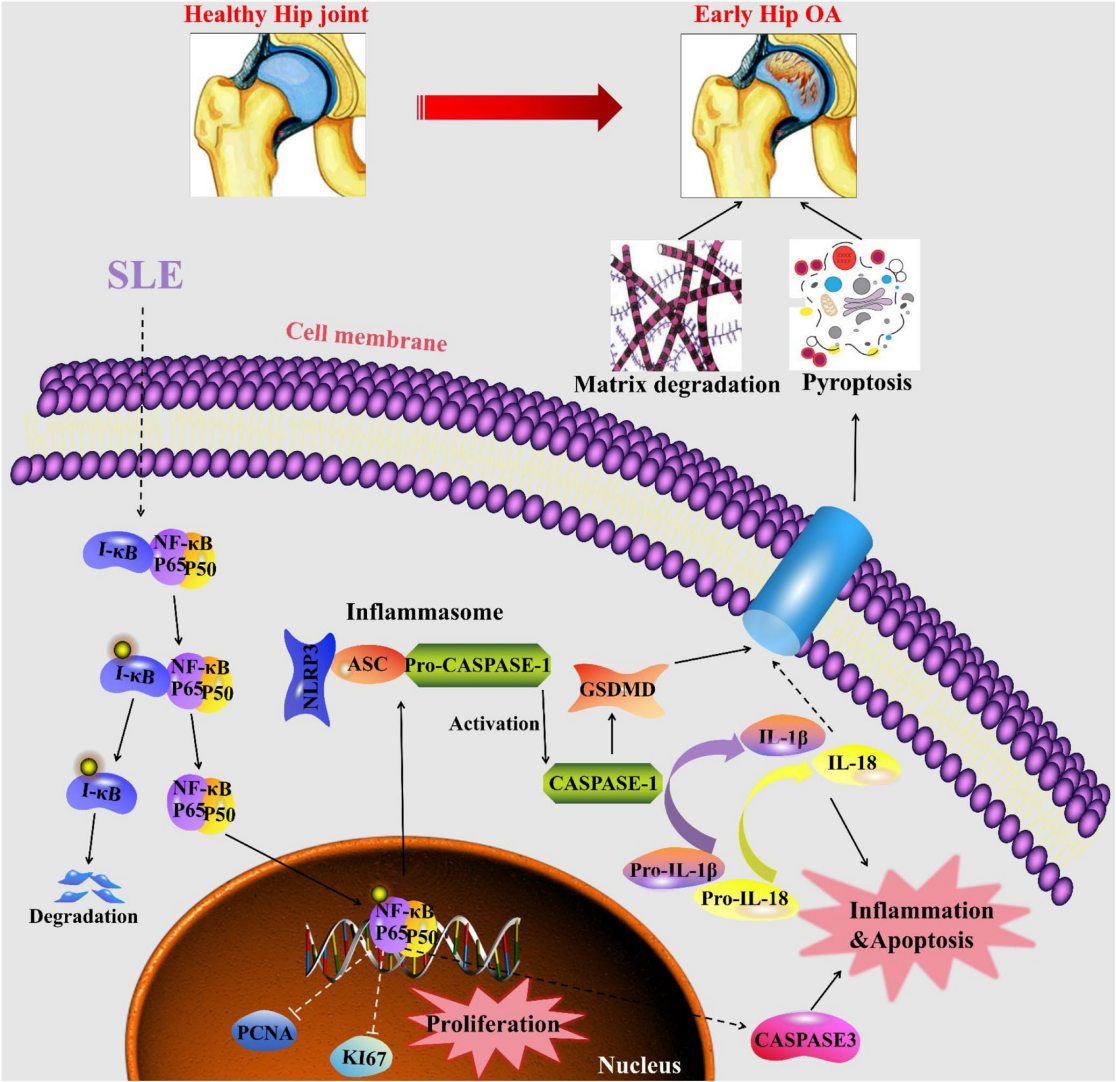
Schematic illustration of SLE‐induced hip arthritis progression. Diagram depicting how SLE promotes hip arthritis development through activation of the NF‐κB pathway‐mediated chondrocyte pyroptosis, leading to inflammatory cytokine release, ECM degradation and cartilage destruction.

SLE is a multifaceted autoimmune disease characterised by chronic inflammation and immune dysregulation, with joint involvement being one of its earliest and most devastating manifestations [40]. Clinical studies reveal that 81.7% of SLE patients experience joint symptoms and 86.7% report joint pain, with a particularly concerning trend showing an increasing need for early hip replacement surgery due to accelerated joint destruction [7]. Despite this high prevalence and significant impact on patients' quality of life, only 26.8% of cases are diagnosed early enough to prevent severe joint damage [41, 42]. This delayed intervention, combined with the fact that 63%–93% of SLE patients develop secondary arthritis, makes hip arthritis one of the most severe complications of SLE [43, 44]. While the clinical burden of SLE‐associated hip arthritis is well documented [45, 46], the molecular mechanisms driving this accelerated joint destruction have remained elusive. In this study, we demonstrate that SLE triggers a complex cascade of pathological events in the hip joint cartilage of MRL/*lpr* mice, characterised by impaired chondrocyte proliferation, enhanced apoptosis and amplified inflammatory responses. Most significantly, we identified that SLE activates chondrocyte pyroptosis through NF‐κB pathway stimulation, leading to increased expression of NLRP3, ASC, CASPASE‐1 and GSDMD. These findings establish pyroptotic cell death as a central mechanism linking SLE to accelerated cartilage destruction, suggesting that targeting this pathway could provide therapeutic benefits for protecting joint integrity in SLE patients (Figure 6).

MRL/*lpr* lupus‐prone mice, with accelerated autoimmune responses caused by *Fas* (CD95) mutations, are widely recognised as a robust model for studying human autoimmune diseases and their associated complications. These mice exhibit hallmark pathological features of SLE, including lupus nephritis, autoantibody production, cutaneous manifestations, lymphadenopathy, splenomegaly and haematological abnormalities [47, 48, 49]. In addition, MRL/*lpr* mice also display significant musculoskeletal involvement, including arthritis, joint inflammation and AC degeneration, making them a valuable tool for investigating the mechanisms of SLE‐associated arthritis and joint damage [47, 48, 49]. Joint symptoms, ranging from intermittent joint pain to acute polyarthritis, occur in approximately 90% of SLE patients, with many cases progressing to severe secondary arthritis [50, 51]. Consistent with these findings, our study investigated the impact of SLE on hip joint homeostasis in MRL/*lpr* mice. We found that the onset of SLE triggers AC degeneration in the femoral heads, as evidenced by structural damage, disrupted ECM metabolism, chondrocyte loss and an intensified inflammatory response. These findings provide new insights into the pathological interplay between SLE and hip arthritis progression, emphasising the role of SLE‐induced inflammation and immune dysregulation in exacerbating joint degeneration.

Extensive research has shown that SLE promotes the congregation of lupus autoantigens inside apoptotic cells and induces overproduction of inflammatory cytokines in various tissues, significantly influencing the development of SLE‐related manifestations such as kidney damage [52] and cardiovascular complications [53, 54]. Elevated serum IL‐1β levels have been closely linked to the severity of lupus‐associated arthritis, highlighting its pivotal role in inflammatory responses and tissue injury, while our latest findings suggest that the onset of SLE triggers excessive inflammation in the nucleus pulposus, a chondrocyte‐like tissue in the intervertebral disc, thereby facilitating the progression of intervertebral disc degeneration [17]. In our study, SLE notably enhanced cytokine production in the femoral head, with IL‐1β levels increasing by 5.1‐fold. This inflammatory cytokine surge was associated with ECM degradation and chondrocyte apoptosis, contributing to AC degeneration and the progression of hip arthritis‐like conditions. Unlike apoptosis, which generally does not provoke a strong inflammatory response, pyroptosis operates more rapidly and is accompanied by the substantial release of pro‐inflammatory factors [55]. These observations indicate that SLE‐associated hip arthritis‐like progression is likely driven by pyroptosis‐mediated excessive cytokine production, linking heightened inflammatory activity to cartilage deterioration.

Pyroptosis is a key driver of the inflammatory response, characterised by the release of cytokines IL‐1β and IL‐18 [56, 57]. Studies have extensively documented the role of chondrocyte pyroptosis in femoral head inflammation, which accelerates hip arthritis progression, while targeting pyroptosis has thus emerged as a potential therapeutic approach to delay AC degeneration [39, 58]. Additionally, research has shown that SLE triggers NLRP3‐mediated pyroptosis in the intervertebral disc, kidneys, spleen and thymus of MRL/*lpr* mice, thereby worsening SLE pathogenesis and related complications [59, 60, 61]. Consistent with these observations, our results demonstrate that SLE significantly induces chondrocyte pyroptosis within the hip joints of MRL/lpr mice, indicated by increased expression of NLRP3, ASC, CASPASE‐1 and GSDMD. These findings suggest that pyroptosis, rather than apoptosis, plays a dominant role in driving SLE‐induced hip arthritis by triggering inflammatory cascades and exacerbating AC degeneration.

NF‐κB signalling is a well‐established driver of arthritis pathophysiology, promoting inflammation and upregulating proteins involved in ECM catabolism [62]. Recent studies, including those from our team, have demonstrated that dysregulated NF‐κB activation induces chondrocyte pyroptosis, contributing to chondrocyte loss and accelerated ECM breakdown [39, 58, 63, 64, 65, 66, 67, 68]. However, the specific role of the NF‐κB pathway in chondrocyte pyroptosis during hip arthritis progression remains unclear. Herein, we identified that SLE deterioration led to a notable increase in P65 protein and phosphorylation of i‐κB, two key proteins in the NF‐κB pathway. These findings suggest that SLE‐triggered NF‐κB activation may drive chondrocyte pyroptosis, leading to ECM degradation, chondrocyte depletion, heightened inflammation and AC damage, thereby contributing to the progression of hip arthritis.

Our research was prompted by three critical challenges in the field: the high prevalence of SLE‐associated arthritis, the paucity of efficacious therapeutic interventions and the clinical underemphasis on early detection strategies. The findings presented herein have significant translational implications for both pharmacological development and diagnostic advancement. Our results have highlighted the potential pathogenesis linking SLE to the exacerbation of AC degeneration and the progression of hip arthritis. Specifically, we demonstrate that chondrocyte pyroptosis serves as a pivotal molecular nexus through which excessive pro‐inflammatory cytokine production drives and perpetuates joint destruction in SLE. This mechanistic insight identifies the NF‐κB/pyroptosis axis as a promising therapeutic target for developing selective anti‐inflammatory agents with the potential to mitigate articular damage in SLE patients. Additionally, our utilisation of the MRL/*lpr* murine model, characterised by its accelerated disease phenotype, provides a valuable experimental platform for identifying early biomarkers of joint involvement. These findings may facilitate the development of predictive diagnostic criteria for arthritis in SLE patients who are either in preclinical stages or presenting with subclinical joint manifestations, potentially enabling preventive interventions before irreversible structural damage occurs.

While this study provides valuable insights, several limitations must be acknowledged. First, the exclusive use of the MRL/*lpr* mouse model limits the generalisability of the findings, necessitating validation in other lupus‐prone models like NZB/W F1 mice [69]. Second, the focus on 14‐week‐old mice captures only short‐term effects, overlooking the chronic and progressive nature of both SLE and hip arthritis. Long‐term studies on older mice are essential to better understand disease dynamics. Third, translating these findings to humans requires validation with clinical samples from SLE patients to confirm the relevance of the observed mechanisms. Lastly, while pyroptosis‐related proteins were examined, the causal role of chondrocyte pyroptosis in hip arthritis progression was not directly validated. Future research using specific inhibitors or genetic models targeting pyroptosis pathways will be crucial to address this gap and enhance the translational impact of the findings.

## 5 Conclusion

To summarise, our study demonstrates that SLE accelerates AC degeneration in SLE‐associated arthritis through multiple pathological mechanisms, with pyroptosis emerging as a central mediator. Our findings reveal that SLE not only impairs chondrocyte proliferation and promotes apoptosis but crucially triggers NLRP3‐mediated pyroptosis via NF‐κB pathway activation, thereby triggering a cascade of excessive inflammatory cytokine release and ECM degradation that specifically perpetuates joint damage in SLE‐associated arthritis. These discoveries establish NLRP3‐mediated pyroptosis as a central mechanism driving cartilage destruction in the context of SLE inflammatory joint disease, offering potential therapeutic targets for mitigating AC degeneration and alleviating hip arthritis progression specifically in SLE patients.

## Author Contributions

**Xuliang Fang:** formal analysis (equal), investigation (equal), software (equal), validation (equal), writing – original draft (equal), writing – review and editing (equal). **Helou Zhang:** investigation (equal), software (equal), validation (equal), writing – original draft (equal), writing – review and editing (equal). **Huiqing Zhou:** validation (equal), writing – review and editing (equal). **Shuchao Shen:** investigation (equal), validation (equal). **Zhaobai Lao:** investigation (equal), validation (equal), writing – review and editing (equal). **Zhiguo Zhang:** validation (equal), writing – review and editing (equal). **Yishan Bian:** validation (equal), writing – review and editing (equal). **Chengcong Zhou:** validation (equal), writing – review and editing (equal). **Hongting Jin:** data curation (equal), supervision (equal). **Peijian Tong:** data curation (equal), supervision (equal). **Yanqun Huang:** data curation (equal), funding acquisition (equal), supervision (equal). **Hong Zhou:** data curation (equal), funding acquisition (equal), supervision (equal). **Hanbing Zeng:** data curation (equal), funding acquisition (equal), supervision (equal). **Fangda Fu:** data curation (equal), funding acquisition (equal), methodology (equal), supervision (equal). **Chengliang Wu:** conceptualization (equal), funding acquisition (equal), writing – review and editing (equal). **Wenbiao Zheng:** data curation (equal), formal analysis (equal), supervision (equal), writing – review and editing (equal). **Hongfeng Ruan:** conceptualization (equal), funding acquisition (equal), methodology (equal), writing – original draft (equal), writing – review and editing (equal).

## Conflicts of Interest

The authors declare no conflicts of interest.

## Data Availability

The data that support the findings of this study are available from the corresponding author upon reasonable request.
